# Consensus recommendations for the diagnosis and treatment of acquired hemophilia A

**DOI:** 10.1186/1756-0500-3-161

**Published:** 2010-06-07

**Authors:** Peter Collins, Francesco Baudo, Angela Huth-Kühne, Jørgen Ingerslev, Craig M Kessler, Maria E Mingot Castellano, Midori Shima, Jean St-Louis, Hervé Lévesque

**Affiliations:** 1 Arthur Bloom Hemophilia Centre, School of Medicine, Cardiff University, University Hospital of Wales, Cardiff, CF14 4XN, UK; 2 Thrombosis and Hemostasis Unit, Niguarda Hospital, I-20162 Milan, Italy; 3 SRH Kurpfalzkrankenhaus Heidelberg GmbH and Hemophilia Centre, D-69123 Heidelberg, Germany; 4 Centre for Hemophilia and Thrombosis, Skejby University Hospital, Department of Clinical Biochemistry, DK-8200 Aarhus, Denmark; 5 Department of Medicine and Pathology, Division of Hematology/Oncology and Georgetown University Hospital, Lombardi Cancer Center, Division of Hem/Onc, Washington, DC 20057, USA; 6 Regional Universitary Hospital Carlos Haya, Division of Hematology, E-29004 Málaga, Spain; 7 Department of Pediatrics, Nara Medical University, 634-8522 Nara, Japan; 8 Department of Medicine, Université de Montréal and Hématologie-Oncologie, Hôpital Maisonneuve-Rosemont, Montréal, QC H1T 2 M4, Canada; 9 Department of Internal Medicine, Centre Hospitalier Universitaire de Rouen-Boisguillaume, F- 76031 Rouen, France

## Abstract

**Background:**

Acquired hemophilia A (AHA) is a rare bleeding disorder caused by an autoantibody to coagulation factor (F) VIII. It is characterized by soft tissue bleeding in patients without a personal or family history of bleeding. Bleeding is variable, ranging from acute, life-threatening hemorrhage, with 9-22% mortality, to mild bleeding that requires no treatment. AHA usually presents to clinicians without prior experience of the disease, therefore diagnosis is frequently delayed and bleeds under treated.

**Methods:**

Structured literature searches were used to support expert opinion in the development of recommendations for the management of patients with AHA.

**Results:**

Immediate consultation with a hemophilia center experienced in the management of inhibitors is essential to ensure accurate diagnosis and appropriate treatment. The laboratory finding of prolonged activated partial thromboplastin time with normal prothrombin time is typical of AHA, and the diagnosis should be considered even in the absence of bleeding. The FVIII level and autoantibody titer are not reliable predictors of bleeding risk or response to treatment. Most patients with AHA are elderly; comorbidities and underlying conditions found in 50% of patients often influence the clinical picture. Initial treatment involves the control of acute bleeding with bypassing agents. Immunosuppressive treatment to eradicate the FVIII inhibitor should be started as soon as the diagnosis is confirmed to reduce the time the patient is at risk of bleeding.

**Conclusions:**

These recommendations aim to increase awareness of this disorder among clinicians in a wide range of specialties and provide practical advice on diagnosis and treatment.

## Background

Acquired hemophilia A (AHA) is a bleeding disorder caused by an autoantibody to factor VIII [1-4]. It must be distinguished from congenital hemophilia, which is an inherited disorder caused by mutations in the FVIII gene that presents at a young age and is characterized by a distinct bleeding phenotype. AHA has an estimated incidence of 1.5/million/year and predominantly affects older patients [5-7]. The disorder presents with bleeding, ranging from life- and limb-threatening to mild in patients with no personal or family history of bleeding, and has a high mortality, estimated at between 9-22% [5,8].

Patients usually present to physicians who are not specialists in the field and have not previously managed a case, typically geriatricians, obstetricians, rheumatologists, oncologists, emergency physicians, intensive care physicians or surgeons as well as hematologists. Lack of familiarity with the disorder may lead to delayed diagnosis and suboptimal treatment, therefore immediate consultation with a hemophilia centre experienced in the management of inhibitors is required, irrespective of the clinical features at presentation [9,10]. The clinical phenotype does not correlate with the factor VIII level or inhibitor titer [1,5], and patients remain at risk of spontaneous, life-threatening bleeding until the inhibitor has been eradicated [5], even if the initial presentation is associated with mild or no bleeding. The age of the patients and associated comorbidities [11] often influence the clinical phenotype and the risk of treatment-induced side effects [1].

There is no high-level evidence to support management recommendations for patients with AHA. Some data generated in patients with congenital hemophilia can be used to support treatment decisions, however, most treatment recommendations rely on the clinical experience of physicians who have managed patients with the disorder [10]. This guideline is intended to increase the awareness of this rare but often fatal disorder among health care professionals to whom patients with AHA usually present. Clinicians who require more detailed management guidelines are referred to other publications [10,12].

## Methods

Literature searches were performed using the indexed online databases MEDLINE/PubMed using the terms "acquired h(a)emophilia", "acquired factor VIII inhibitors, "acquired inhibitors" and "h(a)emophilia with inhibitors". The full manuscripts from relevant abstracts were retrieved and supplemented by literature from the authors' own libraries. Approximately 150 articles were retrieved and distributed to the author group. The manuscript was written by the authors, coordinated and supported by Physicians World Europe GmbH, Mannheim, Germany. Recommendations were formulated according to the method of Guyatt et al. [13], where "we recommend" represents a strong (Grade 1) recommendation and "we suggest" a weak (Grade 2) recommendation. Because no high or medium-level evidence exists to support management recommendations, the literature cited was not graded according to level of evidence. A parallel manuscript for specialist physicians was also developed [12].

The authors comprise an independent, international medical collaboration with expertise in the field of AHA. The need for increased awareness of the disorder and practice-based guidelines was initially suggested by one member of the author group (CK). The group's activities were funded by unrestricted educational grants from Novo Nordisk Health Care AG, Zurich, Switzerland, a company that commercializes recombinant factor VIIa (NovoSeven^®^), an agent manufactured by Novo Nordisk A/S, Bagsvaerd, Denmark and used to treat bleeding in AHA.

## Results and Discussion

### Diagnosis

#### Clinical presentation

**Figure 1:**
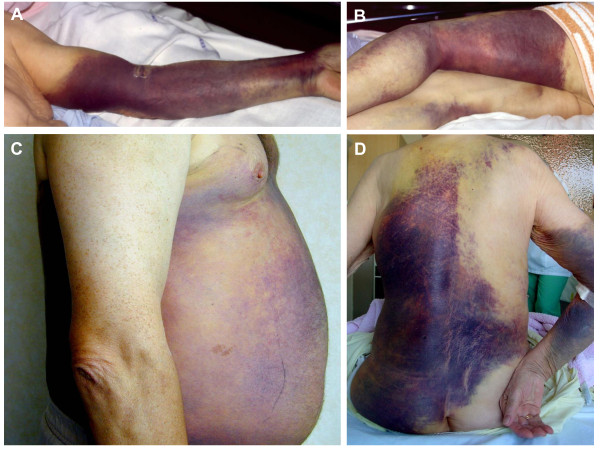
**Extensive subcutaneous ecchymoses of the limbs, thorax and abdomen**.

Patients with AHA tend to be elderly, with a median age of 77 years [11]. The incidence per million per year increases with age from 0.3 in 16-64 year olds to 9 in 65-84 year olds and 15 in those aged 85 and older [5]. Patients with AHA present with a bleeding pattern that is distinct from congenital hemophilia. Severity in congenital hemophilia can be predicted from the factor VIII level, and patients predominantly experience hemarthroses, trauma-induced muscle bleeds and other soft tissue bleeds. AHA patients typically present with widespread subcutaneous bleeds (seen in 80% of cases) and other soft tissue and mucosal bleeding such as muscle hematoma, urinogenital tract bleeding (including post partum) and other mucosal sites. Compartment syndromes associated with neurovascular injury are also seen, however hemarthroses are uncommon (Tables 1 &2). Bleeds in AHA are often spontaneous, and the severity does not correlate with the factor VIII level [3] or strength of the inhibitor. Severe bleeding following invasive procedures is almost inevitable, and venipuncture commonly results in extensive subcutaneous bleeding. Fatal hemorrhage occurs in about 9-22% of cases [5,8]. In contrast, mild bleeding, requiring no haemostatic treatment, is seen in about 30% of cases [1,5,8,14,15] (Figure 1). Patients occasionally present with abnormal routine blood tests without clinical evidence of bleeding.

**Table 1:** Characteristic symptoms associated with acquired hemophilia.

-	Acute onset of severe and life-threatening bleeding or widespread subcutaneous bleeds
-	Bleeding sites atypical of congenital hemophilia
-	High mortality with both early and late deaths
-	Presence of underlying diseases and conditions
-	Advanced age

**Table 2:** The distribution of bleeding symptoms in two cohorts of patients^a ^with AHA.

Type of bleeding	**Collins et al. 2007 **[5] (%)	**Morrison et al. 1993 **[14] (%)
	N = 172	N = 65
Subcutaneous/skin	81	23
Muscle	45	32
Subcutaneous only	24	
Gastrointestinal/intrabdominal	23	14
Genital urinary	9	18
Retroperitoneal/thoracic	9	5
Other	9	23
Post-operative		11
Joint	7	2
None	4	
Intracranial hemorrhage	3	
Fatal	9	
No haemostatic treatment required	34	

^a^In the study by Collins et al. [5] all bleeds at presentation were recorded. In the study by Morrison et al. [14] bleeds requiring treatment were recorded. The studies are therefore not directly comparable.

Patients remain at risk of fatal bleeding until the inhibitor has been eradicated, even if they initially present with mild or no bleeding [5]. Early hemorrhagic deaths, within the first week, may result from gastrointestinal or lung bleeding, whereas later deaths are predominantly due to soft tissue bleeding such as intracranial and retroperitoneal bleedings [5].

In about half of patients, an underlying condition is observed (Table 3). Autoimmune diseases, including rheumatoid arthritis and systemic lupus erythematosus, polymyalgia rheumatica, malignancies, dermatologic disease (especially pemphigoid) and pregnancy are common [5,8,14]. Associated medical conditions and treatment may contribute to the clinical presentation and response to treatment.

**Table 3:** Disease states associated with acquired hemophilia.

	Green, 1981 [8]	**Morrison, 1993 **[14]	**Collins, 2007 **[5]
N	215	65	150
Idiopathic (%)	46	55	63
Collagen, vascular, and other autoimmune diseases (%)	18	17	17
Malignancy (%)	7	12	15
Skin diseases (%)	5	2	3
Possible drug reaction (%)	6	3	0
Pregnancy (%)	7	11	2

**We recommend that physicians managing a patient with suspected or confirmed acquired hemophilia A, with or without bleeding, consult a hemophilia centre with expertise in managing inhibitors as soon as possible**.

AHA requires specialist clinical and laboratory expertise and facilities for diagnosis and treatment [9,10]. Transfer to a specialist centre is often appropriate. If consultation with or transfer to a hemophilia centre is not immediately possible then investigation and treatment should be initiated while a liaison is being established.

#### Laboratory investigation

**We recommend that a diagnosis of acquired hemophilia A be considered in all patients with recent onset of bleeding and/or an unexplained prolonged activated partial thromboplastin time with a normal prothrombin time**.

**Figure 2:**
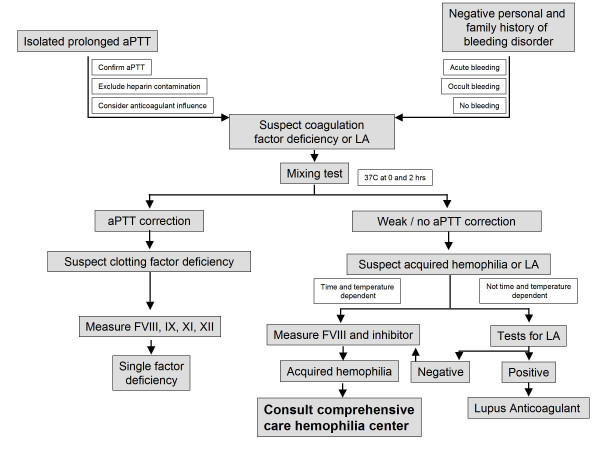
**Algorithm to guide the management of patients with suspected acquired hemophilia**. AH, acquired hemophilia; LA, lupus anticoagulant; F, coagulation factor; aPTT, activated partial thromboplastin time.

**We suggest use of an algorithm such as that shown in Figure **2** for the differential diagnosis of an isolated prolonged activated partial thromboplastin time (aPTT)**.

The diagnosis of AHA is suggested by the clinical picture and confirmed by laboratory investigation. First-line investigations for a patient with unexplained bleeding include a full blood count to assess platelet number and a coagulation screen. The typical finding in patients with AHA is a prolonged aPTT with a normal prothrombin time (PT).

#### Investigation of an isolated prolonged activated partial thromboplastin time

A prolonged aPTT with a normal PT may be due to a deficiency of one of the intrinsic coagulation factors (FVIII, IX, XI or XII) or indicate the presence of an inhibitor. An inhibitor may be against one of the intrinsic factors, most commonly FVIII, but may also be a lupus anticoagulant. Tests for a lupus anticoagulant will be normal in the presence of a FVIII inhibitor, but a lupus anticoagulant may lead to artefactually low factor VIII levels and therefore mimic AHA [16].

Irrespective of the presence or absence of bleeding, an isolated prolonged aPTT outside the normal range should be investigated further. If a patient has bleeding suggestive of AHA further investigation is required even if the aPTT is normal. Some prolonged aPTTs are clinically insignificant but must be adequately investigated to exclude an intrinsic factor deficiency or an inhibitor before this conclusion can be drawn.

#### Mixing tests

To differentiate between an inhibitor and a factor deficiency, mixing studies with normal plasma are used. In these studies patient plasma is mixed with pooled normal plasma in a ratio of 1:1. If the patient plasma has a prolonged aPTT secondary to a deficiency of a clotting factor then the normal plasma will provide the missing factor and the aPTT will be corrected to normal. If on the other hand a patient has an inhibitor to factor VIII, then the factor VIII in the normal plasma will be inhibited and the aPTT will not correct. Failure of normal plasma to correct the aPTT by more than 50% is usually taken as evidence that an inhibitor is present [17], although in some laboratories other definitions are used, and there is no international consensus. FVIII inhibitors, however, are time- and temperature-dependent because the inhibitor does not inhibit the factor VIII in the normal plasma immediately. Therefore, mixing studies must be incubated for 1-2 h at 37°C [10,18]. A mixing study performed without incubation for 1-2 hours or at a lower temperature may result in correction of the aPTT and the diagnosis of AHA potentially missed. Correction of the aPTT with normal plasma does not exclude a FVIII inhibitor, and if the clinical picture is suggestive of AHA, further investigation is warranted. If mixing tests are compatible with an inhibitor, or the clinical picture is suggestive of AHA, the sample should be urgently referred to a specialist hemostasis laboratory for further investigation by Bethesda assay to measure the strength of the factor VIII inhibitor [19].

#### Treatment

The principles of treating AHA are to control bleeding, avoid procedures that may induce bleeds, initiate immunosuppression to eradicate the inhibitor and treat any underlying disease [3,4,9,10,20].

**We recommend that invasive diagnostic or therapeutic procedures not be undertaken unless essential and cannot be delayed. Invasive procedures should be undertaken at a hemophilia centre experienced in the treatment of inhibitors**.

Patients with AHA should not be subjected to invasive procedures, unless these are essential and cannot be delayed, because uncontrollable bleeding may result from even minor procedures. Venipuncture, the placement of a venous cannula and measurement of blood pressure may also lead to severe bleeding and should be kept to a minimum. Lumbar puncture should not be performed without haemostatic cover. Intramuscular injections are contraindicated. Unavoidable invasive procedures such as the placement of central lines, arterial puncture for blood gas assessment or lumbar puncture should be performed under cover of a bypassing agent.

#### Treatment of hemorrhage

**We recommend that bleeding episodes in patients with AHA be treated in consultation with a hemophilia centre experienced in the treatment of inhibitors**.

Many patients require immediate treatment to control bleeding, but not all types of bleeding require intervention [5,15]. The cardiovascular comorbidities associated with advanced age may put patients at risk of thrombotic complications during haemostatic therapy. Factor VIII levels or inhibitor titers are of no value in guiding anti-hemorrhagic treatment [3,4,10].

First-line treatment of bleeding in AHA is with a bypassing agent. The two available licensed treatments are recombinant factor VIIa (rFVIIa, NovoSeven^®^) [21,22] and the activated prothrombin complex concentrate (aPCC) (FEIBA^®^; Factor VIII inhibitor bypassing activity) [23]. The treating physician should be experienced in the use of these products [24,25], or should transfer the patient to a centre where this expertise is available. If transfer is not possible, then haemostatic treatment should not be delayed and treatment should be initiated at standard doses while contact with a hemophilia centre is established for advice. Standard treatment with rFVIIa is 90 mcg/kg every 2 h until the bleed has been controlled followed by further, less frequent dosing, to prevent recurrence. FEIBA is dosed at 50-100 U/kg every 8-12 h with a maximum dose of 200 U/kg/day until the bleed has been controlled with additional doses to prevent recurrence.

Data on the efficacy of anti-hemorrhagic treatment are retrospective and include a limited number of patients with a wide range of conditions. Prospective randomized clinical studies that compare the efficacy of the available agents in AHA have not been performed and are impracticable due to the rarity and diverse clinical features of the disorder. A prospective randomized trial of hemarthroses in congenital hemophilia did not demonstrate any difference in efficacy between rFVIIa and FEIBA, although criteria for equivalence were not met [26]. This study must be viewed with caution in the context of AHA because, while hemarthroses are common in congenital hemophilia, they are unusual in AHA. Retrospective studies and clinical experience in AHA show that bypassing agents are effective in about 90% of patients [22,23,26]. In cases where one bypassing agent is ineffective, good efficacy may be achieved with the alternative agent.

The use of bypassing agents in AHA is associated with a risk of arterial thrombosis because many patients have cardiovascular risk factors. A literature review reported that 7% of patients treated with rFVIIa experienced a thrombotic event [22]. The high risk of life-threatening bleeding if bleeds are not treated early and adequately, however, outweighs the risk of thrombosis in most situations, although large subcutaneous bleeds often require no treatment.

Response to haemostatic therapy is usually best assessed clinically and by measurement of the hemoglobin or hematocrit. Radiological imaging sometimes plays a role. There are no laboratory tests that have been demonstrated to be useful for monitoring the efficacy of bypassing agents. After hemostasis has been achieved, further treatment may be required to prevent re-bleeds at the same site.

If the level of the inhibitor is very low and no bypassing agent is available, hemostasis can sometimes be achieved with high doses of FVIII [27]. The response to this treatment, however, is unpredictable and the use of factor VIII should not delay the use of agents more likely to control bleeding. Factor VIII is more efficacious when used as part of multimodal treatment regimens that include immunoadsorption to temporarily remove the inhibitor. However, at the time of writing immunoadsorption columns are not available in all countries, and this therapy is available in only a few highly specialized centers [27-29].

Desmopressin (DDAVP) at a dose of 0.3 mcg/kg intravenously may be useful in the context of minor bleeding episodes and very low titer inhibitors [30-33] but the use of DDAVP should not delay the use of more effective drugs. There is no evidence to support the use of intravenous gammaglobulin as a single agent to resolve bleeding.

#### Treatment to eradicate the inhibitor

**We recommend that all patients diagnosed with acquired hemophilia A receive immunosuppressive therapy immediately following diagnosis**.

The risk of fatal bleeding continues until the factor VIII antibody has been eradicated, therefore immunosuppression should be commenced in all patients as soon as the diagnosis has been made [2]. Immunosuppression is best undertaken in consultation with a hemophilia centre experienced in the management of inhibitors, but if advice is not immediately available, treatment should not be delayed.

The optimal strategy for inhibitor eradication is unknown. The two most common regimens are corticosteroids alone or corticosteroids combined with cyclophosphamide (for reviews see [1,9]). Some experts recommend the combination of steroids and cyclophosphamide as initial treatment, asserting that the inhibitor is eradicated more rapidly [20]. However, the published evidence from retrospective studies suggests that neither strategy leads to a superior outcome [1,5,9] and the only randomized study comparing the two regimens did not recruit sufficient patients to be interpretable [34]. Reviews of published data and large cohorts suggest that about 70-80% of patients achieve remission with steroids alone or steroids combined with cyclosphosphamide but studies have variable treatment regimens and different definitions of endpoints and so cannot be directly compared [1,5,9]. We therefore suggest that immunosuppression be initiated with either prednisolone alone at a dose of 1 mg/kg or a combination of prednisolone and cyclophosphamide (1-2 mg/kg).

The median time to remission with corticosteroid treatment is about 5 weeks [9], however if the factor VIII level has not started to increase and the inhibitor titer decline after 2-3 weeks, an alternative immunosuppressive regimen should be considered. If corticosteroid treatment has been initiated and fails to induce remission, commonly used strategies include the addition of cyclophosphamide [1,9,10,34] or rituximab [1,7,34], although rituximab is not licensed for this indication. Third-line therapies include rituximab [35-37], if not already used, or combinations of cytotoxic agents or cyclosporin [38,39]. The side effects associated with these drugs must be taken into consideration, particularly in the context of patient age [11]. Neutropenia with subsequent infections and sepsis have been reported and may contribute to patient mortality [1,5,14,39-43].

Patients may be monitored on an outpatient basis, except in the presence of bleeding or high comorbidity. Patients should be educated about how to report relevant symptoms and access specialist services on a 24-hour basis. Routine monitoring should include physical examination, hemogram, aPTT, FVIII activity and FVIII inhibitor titer assessments. Relapse is seen in up to 20% of patients, and prolonged follow-up is required [5]. Elevated FVIII levels in AHA patients in remission are common, and appropriate prophylactic management in thrombosis-prone patients should be considered during high risk clinical episodes [44]. Thromboembolic risk in general should be managed as in any other patient without a history of a coagulation disorder [45], although there are no studies into thromboprophylaxis in the context of patients recovering from AHA.

## Conclusions

Because AHA is a rare but often fatal disorder that usually presents to clinicians without previous experience of the disorder there is often a delay in diagnosis and hence under treatment. Due to the high mortality and morbidity associated with this condition and the complexity of diagnosis and treatment, immediate consultation with a hemophilia centre experienced in the management of inhibitors should be initiated whenever AHA, with or without bleeding, is suspected.

The aim of these recommendations is to increase the awareness of this disorder among health care professionals to whom AHA hemophilia patients are likely to present, encourage timely referral to an experienced specialist physician and create a standardized level of care for this patient group.

## List of abbreviations

AHA: acquired hemophilia A; aPCC: activated prothrombin complex concentrate; aPTT: activated partial thromboplastin time; DDAVP: desmopressin; F: coagulation factor; FEIBA: factor VIII inhibitor bypassing activity; PT: prothrombin time; rF: recombinant factor.

## Competing interests

**PC **has received honoraria for consulting or lecturing from Baxter, Novo Nordisk, Bayer and Ipsen, educational grants from Baxter and Bayer and institutional support from Baxter; **FB **has received honoraria for consulting or lecturing from Bayer HealthCare, and Novo Nordisk; **AH-K **has received honoraria for consulting or lecturing from Bayer HealthCare, Novo Nordisk and Wyeth; **JI **has received honoraria for consulting or lecturing from Baxter, Novo Nordisk, Bayer, Wyeth, educational grants from BioVitrum and institutional support from STER, Baxter and BioVitrum; **CMK**'s institution receives research funding for clinical trials on his behalf from Baxter Immuno, Novo Nordisk, Octapharma, Grifols, Genentech and Bayer. He has received honoraria for consulting or lecturing from Wyeth, Octapharma, Bayer, Baxter Immuno, Ipsen and Novo Nordisk; **MEMC **has received honoraria for consulting or lecturing for Novo Nordisk, Grifols, Baxter and Wyeth. No other relevant financial associations are reported by the Thrombosis and Haemostasis section of the Haematology department at the Regional University Hospital of Carlos Haya, Malaga (Spain); **MS **reported no relevant financial associations; **JSL **has received honoraria for consulting from Baxter and Novo Nordisk, educational grants from Bayer and research grants from Ipsen and Novartis; **HL **has received honoraria for consulting or lecturing from Novo Nordisk.

## Authors' contributions

**FB, AH-K, HL, MEMC **and **JSL **were present at one or both of two initial meetings to define the scope of the present manuscript and the published guidelines for specialist physicians. **FB, JI, AH-K, HL, MEMC, MS **and **JSL **were present during the first consensus meeting to present the results of the literature review process.

Three working groups were formed to write each section of both manuscripts; communication occurred electronically or by telephone: diagnosis (**PC, JI, MS**), hemorrhagic treatment (**FB, CMK, JSL**), inhibitor eradication (**HL, AH-K, MEMC**).

**FB, JI, CMK, JSL, AH-K, HL **and **MEMC **were present for the final consensus meeting on both guideline manuscripts. **PC **prepared the final draft of the manuscript, which was reviewed and approved by all authors. The opinions expressed in this manuscript are those of the authors alone. All authors had full access to all of the literature described here and take responsibility for the integrity of the data and the accuracy of the data analysis.

## References

[B1] Delgado J Jimenez-Yuste V Hernandez-Navarro F Villar A Acquired haemophilia: review and meta-analysis focused on therapy and prognostic factors Br J Haematol 2003 121 1 21 35 10.1046/j.1365-2141.2003.04162.x 12670328

[B2] Hay CR Acquired haemophilia Baillieres Clin Haematol 1998 11 2 287 303 10.1016/S0950-3536(98)80049-8 10097808

[B3] Kessler CM Asatiani E Lee CA, Berntorp EE, Hoots WK, Aledort LM Acquired Inhibitors to Factor VIII Textbook of Hemophilia 2007 86 90

[B4] Morrison AE Ludlam CA Acquired haemophilia and its management Br J Haematol 1995 89 2 231 236 10.1111/j.1365-2141.1995.tb03294.x 7873371

[B5] Collins PW Hirsch S Baglin TP Dolan G Hanley J Makris M Keeling DM Liesner R Brown SA Hay CR Acquired hemophilia A in the United Kingdom: a 2-year national surveillance study by the United Kingdom Haemophilia Centre Doctors' Organisation Blood 2007 109 5 1870 1877 10.1182/blood-2006-06-029850 17047148

[B6] Collins P Macartney N Davies R Lees S Giddings J Majer R A population based, unselected, consecutive cohort of patients with acquired haemophilia A Br J Haematol 2004 124 1 86 90 10.1046/j.1365-2141.2003.04731.x 14675412

[B7] Borg JY Levesque H for the SACHA study group Epidemiology and one-year outcomes in patients with acquired hemophilia in France J Thromb Haemost 2007 5 suppl 1 O-M-062

[B8] Green D Lechner K A survey of 215 non-hemophilic patients with inhibitors to Factor VIII Thromb Haemost 1981 45 3 200 203 6792737

[B9] Collins PW Treatment of acquired hemophilia A J Thromb Haemost 2007 5 5 893 900 10.1111/j.1538-7836.2007.02433.x 17461924

[B10] Hay CR Brown S Collins PW Keeling DM Liesner R The diagnosis and management of factor VIII and IX inhibitors: a guideline from the United Kingdom Haemophilia Centre Doctors Organisation Br J Haematol 2006 133 6 591 605 10.1111/j.1365-2141.2006.06087.x 16704433

[B11] Baudo F de Cataldo F Balducci L, Ershler W, de Gaetano G Acquired haemophilia in the elderly Blood Disorders in the Elderly 2007 Cambridge: Cambridge University Press 389 407

[B12] Huth-Kuhne A Baudo F Collins P Ingerslev J Kessler CM Levesque H Castellano ME Shima M St-Louis J International recommendations on the diagnosis and treatment of patients with acquired hemophilia A Haematologica 2009 94 4 566 575 10.3324/haematol.2008.001743 19336751 PMC2663620

[B13] Guyatt G Gutterman D Baumann MH Addrizzo-Harris D Hylek EM Phillips B Raskob G Lewis SZ Schunemann H Grading strength of recommendations and quality of evidence in clinical guidelines: Report from an American College of Chest Physicians task force Chest 2006 129 1 174 181 10.1378/chest.129.1.174 16424429

[B14] Morrison AE Ludlam CA Kessler C Use of porcine factor VIII in the treatment of patients with acquired hemophilia Blood 1993 81 6 1513 1520 8453098

[B15] Lottenberg R Kentro TB Kitchens CS Acquired hemophilia. A natural history study of 16 patients with factor VIII inhibitors receiving little or no therapy Arch Intern Med 1987 147 6 1077 1081 10.1001/archinte.147.6.1077 3109341

[B16] Greaves M Cohen H MacHin SJ Mackie I Guidelines on the investigation and management of the antiphospholipid syndrome Br J Haematol 2000 109 4 704 715 10.1046/j.1365-2141.2000.02069.x 10929019

[B17] Kasper CK Laboratory tests for factor VIII inhibitors, their variation, significance and interpretation Blood Coagul Fibrinolysis 1991 2 suppl 1 7 101773000

[B18] Lossing TS Kasper CK Feinstein DI Detection of factor VIII inhibitors with the partial thromboplastin time Blood 1977 49 5 793 797 856360

[B19] Giles AR Verbruggen B Rivard GE Teitel J Walker I A detailed comparison of the performance of the standard versus the Nijmegen modification of the Bethesda assay in detecting factor VIII:C inhibitors in the haemophilia A population of Canada. Association of Hemophilia Centre Directors of Canada. Factor VIII/IX Subcommittee of Scientific and Standardization Committee of International Society on Thrombosis and Haemostasis Thromb Haemost 1998 79 4 872 875 9569207

[B20] Franchini M Lippi G Acquired factor VIII inhibitors Blood 2008 112 2 250 255 10.1182/blood-2008-03-143586 18463353

[B21] Hay CR Negrier C Ludlam CA The treatment of bleeding in acquired haemophilia with recombinant factor VIIa: a multicentre study Thromb Haemost 1997 78 6 1463 1467 9423795

[B22] Sumner MJ Geldziler BD Pedersen M Seremetis S Treatment of acquired haemophilia with recombinant activated FVII: a critical appraisal Haemophilia 2007 13 5 451 461 10.1111/j.1365-2516.2007.01474.x 17880429

[B23] Sallah S Treatment of acquired haemophilia with factor eight inhibitor bypassing activity Haemophilia 2004 10 2 169 173 10.1046/j.1365-2516.2003.00856.x 14962206

[B24] Novo Nordisk Health Care AG NovoSeven Summary of Product Characteristics 2006

[B25] Baxter Corp FEIBA VH Anti-inhibitor coagulant complex Vapor Heated Summary of Product Characteristics 2005

[B26] Astermark J Donfield SM DiMichele DM Gringeri A Gilbert SA Waters J Berntorp E A randomized comparison of bypassing agents in hemophilia complicated by an inhibitor: the FEIBA NovoSeven Comparative (FENOC) Study Blood 2007 109 2 546 551 10.1182/blood-2006-04-017988 16990605

[B27] Kasper CK Human factor VIII for bleeding in patients with inhibitors Vox Sang 1999 77 Suppl 1 47 48 10.1159/000056716 10529688

[B28] Zeitler H Ulrich-Merzenich G Hess L Konsek E Unkrig C Walger P Vetter H Brackmann HH Treatment of acquired hemophilia by the Bonn-Malmo Protocol: documentation of an in vivo immunomodulating concept Blood 2005 105 6 2287 2293 10.1182/blood-2004-05-1811 15542586

[B29] Freedman J Rand ML Russell O Davis C Cheatley PL Blanchette V Garvey MB Immunoadsorption may provide a cost-effective approach to management of patients with inhibitors to FVIII Transfusion 2003 43 11 1508 1513 10.1046/j.1537-2995.2003.00559.x 14617307

[B30] Muhm M Grois N Kier P Stumpflen A Kyrle P Pabinger I Bettelheim P Hinterberger W Lechner K 1-Deamino-8-D-arginine vasopressin in the treatment of non-haemophilic patients with acquired factor VIII inhibitor Haemostasis 1990 20 1 15 20 2108912 10.1159/000216100

[B31] de la Fuente B Panek S Hoyer LW The effect of 1-deamino 8 D-arginine vasopressin (DDAVP) in a nonhaemophilic patient with an acquired type II factor VIII inhibitor Br J Haematol 1985 59 1 127 131 10.1111/j.1365-2141.1985.tb02972.x 3918556

[B32] Naorose-Abidi SM Bond LR Chitolie A Bevan DH Desmopressin therapy in patients with acquired factor VIII inhibitors Lancet 1988 1 8581 366 10.1016/S0140-6736(88)91169-5 2893182

[B33] Mudad R Kane WH DDAVP in acquired hemophilia A: case report and review of the literature Am J Hematol 1993 43 4 295 299 10.1002/ajh.2830430413 8372811

[B34] Green D Rademaker AW Briet E A prospective, randomized trial of prednisone and cyclophosphamide in the treatment of patients with factor VIII autoantibodies Thromb Haemost 1993 70 5 753 757 8128430

[B35] Wiestner A Cho HJ Asch AS Michelis MA Zeller JA Peerschke EI Weksler BB Schechter GP Rituximab in the treatment of acquired factor VIII inhibitors Blood 2002 100 9 3426 3428 10.1182/blood-2002-03-0765 12384448

[B36] Stasi R Brunetti M Stipa E Amadori S Selective B-cell depletion with rituximab for the treatment of patients with acquired hemophilia Blood 2004 103 12 4424 4428 10.1182/blood-2003-11-4075 14996701

[B37] Franchini M Rituximab in the treatment of adult acquired hemophilia A: a systematic review Crit Rev Oncol Hematol 2007 63 1 47 52 10.1016/j.critrevonc.2006.11.004 17236786

[B38] Sohngen D Specker C Bach D Kuntz BM Burk M Aul C Kobbe G Heyll A Hollmig KA Schneider W Acquired factor VIII inhibitors in nonhemophilic patients Ann Hematol 1997 74 2 89 93 10.1007/s002770050263 9063379

[B39] Lian EC Larcada AF Chiu AY Combination immunosuppressive therapy after factor VIII infusion for acquired factor VIII inhibitor Ann Intern Med 1989 110 10 774 778 2496636 10.7326/0003-4819-110-10-774

[B40] Shaffer LG Phillips MD Successful treatment of acquired hemophilia with oral immunosuppressive therapy Ann Intern Med 1997 127 3 206 209 9245226 10.7326/0003-4819-127-3-199708010-00005

[B41] Lian EC Villar MJ Noy LI Ruiz-Dayao Z Acquired factor VIII inhibitor treated with cyclophosphamide, vincristine, and prednisone Am J Hematol 2002 69 4 294 295 10.1002/ajh.10070 11921026

[B42] Bossi P Cabane J Ninet J Dhote R Hanslik T Chosidow O Jouan-Flahault C Horellou MH Leynadier F Liozon E Acquired hemophilia due to factor VIII inhibitors in 34 patients Am J Med 1998 105 5 400 408 10.1016/S0002-9343(98)00289-7 9831424

[B43] Huang YW Saidi P Philipp C Acquired factor VIII inhibitors in non-haemophilic patients: clinical experience of 15 cases Haemophilia 2004 10 6 713 721 10.1111/j.1365-2516.2004.01031.x 15569166

[B44] Onitilo AA Skorupa A Lal A Ronish E Mercier RJ Islam R Lazarchick J Rituximab in the treatment of acquired factor VIII inhibitors Thromb Haemost 2006 96 1 84 87 16807656 10.1160/TH06-03-0183

[B45] Geerts WH Pineo GF Heit JA Bergqvist D Lassen MR Colwell CW Ray JG Prevention of venous thromboembolism: the Seventh ACCP Conference on Antithrombotic and Thrombolytic Therapy Chest 2004 126 3 Suppl 338S 400S 10.1378/chest.126.3_suppl.338S 15383478

